# Improving postal survey response using behavioural science: a nested randomised control trial

**DOI:** 10.1186/s12874-021-01476-7

**Published:** 2021-12-18

**Authors:** Emily McBride, Hiromi Mase, Robert S. Kerrison, Laura A. V. Marlow, Jo Waller

**Affiliations:** 1 Department of Behavioural Science and Health, Institute of Epidemiology and Health Care, University College London (UCL), London, UK; 2 Department of Epidemiology and Public Health, Institute of Epidemiology and Health Care, University College London (UCL), London, UK; 3 School of Health Sciences, University of Surrey, Surrey, UK; 4 Cancer Prevention Group, School of Cancer and Pharmaceutical Sciences, King’s College London (KCL), London, UK

**Keywords:** RCT, Behavioural science, Postal response, Methodology, Recruitment, Trials

## Abstract

**Background:**

Systematic reviews have identified effective strategies for increasing postal response rates to questionnaires; however, most studies have isolated single techniques, testing the effect of each one individually. Despite providing insight into explanatory mechanisms, this approach lacks ecological validity, given that multiple techniques are often combined in routine practice.

**Methods:**

We used a two-armed parallel randomised controlled trial (*n* = 2702), nested within a cross-sectional health survey study, to evaluate whether using a pragmatic combination of behavioural science and evidenced-based techniques (e.g., personalisation, social norms messaging) in a study invitation letter increased response to the survey, when compared with a standard invitation letter. Participants and outcome assessors were blinded to group assignment. We tested this in a sample of women testing positive for human papillomavirus (HPV) at cervical cancer screening in England.

**Results:**

Overall, 646 participants responded to the survey (response rate [RR] = 23.9%). Logistic regression revealed higher odds of response in the intervention arm (*n* = 357/1353, RR = 26.4%) compared with the control arm (*n* = 289/1349, RR = 21.4%), while adjusting for age, deprivation, clinical site, and clinical test result (aOR = 1.30, 95% CI: 1.09–1.55).

**Conclusion:**

Applying easy-to-implement behavioural science and evidence-based methods to routine invitation letters improved postal response to a health-related survey, whilst adjusting for demographic characteristics. Our findings provide support for the pragmatic adoption of combined techniques in routine research to increase response to postal surveys.

**Trial registration:**

ISRCTN, ISRCTN15113095. Registered 7 May 2019 – retrospectively registered.

**Supplementary Information:**

The online version contains supplementary material available at 10.1186/s12874-021-01476-7.

## Background

One of the most common data collection methods used in health research is the provision of postal questionnaires, especially when seeking information from large geographically dispersed populations [1]. Postal response rates are considered an important indicator of study quality as they can act as a metric of sample representativeness [1, 2]. Sufficiently high response rates help to reduce some forms of non-response bias, maximise sample size, and minimise research costs [2, 3]. However, adequate response rates are increasingly difficult to obtain with declining rates of participation in health research observed over time, worldwide [4–6].

Systematic reviews have identified effective strategies to increase postal response rates in randomised controlled trials [1, 7–11]. Providing incentives (money, gifts, or prize draws), pre-notifying participants, incorporating university sponsorship, using personalised messages, and sending reminders or a second copy of a questionnaire to non-respondents, have all been shown to increase participant response [1, 8, 12–14]. The design of a questionnaire (content, length, format) can also be altered to achieve differential effects [1, 7, 9]. However, variable effects have been found for observational studies [11].

Dillman’s Tailored Design Method, established in 1970s, has been one of the most common frameworks employed to design research surveys and optimise response rate [15, 16]. More recently, theoretically driven behavioural science techniques have been tested in empirical studies in an attempt to improve participant engagement and response [17–21]. Behavioural frameworks can act as tools for guiding and implementing applied techniques to inform content and design of written materials, such as letters and postal packaging. The ‘MINDSPACE’ Report [22], for example, contains a behavioural science checklist which outlines nine influences on behaviour which can be targeted in routine communications: (i) messenger (we are heavily influenced by who communicates information); (ii) incentives (responses to incentives are shaped by predictable mental shortcuts); (iii) norms (we are strongly influenced by what others do); (iv) default (we tend to follow pre-set options); (v) salience (our attention is drawn to what is novel and seems relevant); (vi) priming (we are influenced by sub-conscious cues); (vii) affect (emotional associations shape our actions); (viii) commitment (we seek to be consistent with public promises and reciprocate acts); and (ix) ego (we act in ways that make us feel better about ourselves). MINDSPACE and other behavioural frameworks are often implemented within research teams and government agencies [21–23]. However, there is a paucity of evidence relating to their efficacy for improving survey response in health research.

Furthermore, most studies aimed at modifying postal response rates have isolated single techniques, testing the effect of each one individually [1, 7–11]. Though these studies provide insight into specific explanatory mechanisms, they lack ecological validity when compared with routine practice, where multiple techniques are often combined. Also, more generally, the behavioural science literature suggests combining relevant behaviour change techniques to maximise effect sizes [24, 25].

The aim of this nested randomised controlled trial (RCT) was to evaluate whether using a pragmatic combination of behavioural science and evidenced-based techniques in a study invitation letter increased response rate to a health-related survey, when compared with a standard invitation letter. We hypothesised that the intervention letter would increase participant return of the postal survey.

## Methods

The manuscript was written in line with CONSORT and TIDieR guidelines – see Supplementary Files 1 and 2, respectively, for completed checklists.

### Design

A two-armed parallel RCT was nested in a cross-sectional psychological survey study of women attending cervical cancer screening in England. The nested RCT aimed to test whether an invitation letter, informed by behavioural science and evidence-based techniques (intervention), increased participant response to a survey, when compared with a standard invitation letter (control).

### Participants, recruitment, and trial setting

Women aged 24 to 66, who had tested HPV-positive with normal cytology at cervical cancer screening for the first or second or third consecutive time, were recruited through two large National Health Service (NHS) clinical sites in England (NHS North London and NHS Greater Manchester). Participants who completed a survey and mailed it back to University College London (UCL) were classified as respondents, while those who did not return a mailed survey were classified as non-respondents. Recruitment occurred between 17.04.2019 and 24.01.2020.

### Randomisation

Simple randomisation of participants was applied in a 1:1 ratio.

### Allocation concealment

Trial arm allocation sequence was determined using a computerised generated random number table [26], which ensured concealment of allocation sequence until the moment of trial arm assignment.

### Implementation

Randomisation was applied by external researchers who were employed within each NHS trust to implement recruitment procedures. These external researchers organised the mailing of the surveys in each of the trial arms.

### Blinding

The researchers who implemented randomisation procedures were blinded to the study objectives. Although participants were exposed to the invitation letter they received, they were unaware that they were part of the nested trial. The data analyst was blinded from group allocation until after statistical analyses had been performed.

### Procedures

Research staff, who were external to the core team, assessed potential participants for eligibility and implemented the recruitment and randomisation procedures at the two recruitment sites. Eligible participants were allocated a unique study identifier by the external researchers, which was used to link pseudonymised survey and clinical data. Names and home addresses of eligible participants by group allocation were uploaded to a secure printing and mailing company (Docmail Ltd) who printed and mailed out the invitation packs (cover letter, information sheet, survey, and pre-paid return envelope). See Supplementary File 3 for the survey used. Potential participants had to return their completed survey to UCL using a pre-paid envelope. To maximise response rate, a reminder pack with the same documents (including the same cover letter) was mailed three weeks later. Some data was recorded directly from clinical records and transferred to UCL for all potential participants, including age, screening test result, NHS site, and Index of Multiple Deprivation score and Quintile (IMD; a multidimensional marker of area-level deprivation based on residential postcode, with quintiles based on national distributions [27]).

### Study materials

**Fig. 1:**
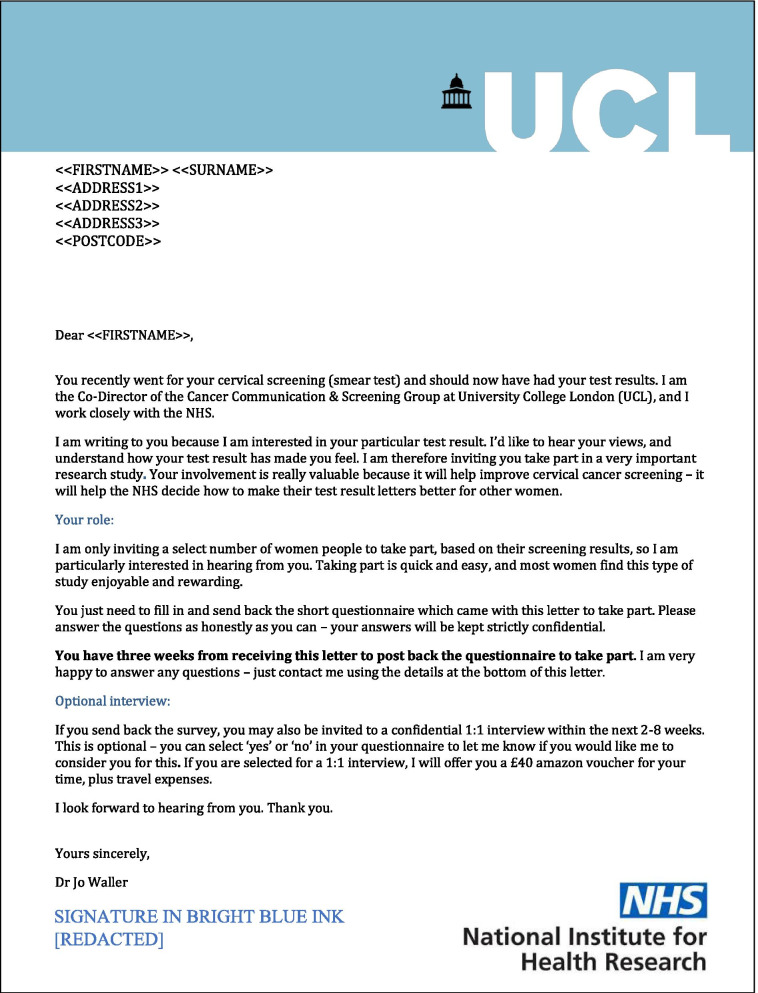
Cover letter used for the intervention group

Participants in the intervention and control groups received the same questionnaire pack and information sheet; however, the cover letter which enclosed these documents differed. The intervention letter (see Fig. 1) employed a combination of techniques expected to improve response rate based on systematic review evidence [8, 9] and the applied behavioural science literature (MINDSPACE [22]). MINDSPACE was chosen as the behavioural science framework to guide intervention letter design because it is commonly used within UK research and policy settings and is comprehensive, bringing together several behavioural science theories in an applied format. Table 1 provides a summary of the techniques used in the intervention letter.

**Table 1:** Summary of techniques used in the intervention letter

**Technique**	**Example detailed in the intervention letter**	**Dominant Rationale or Theoretical Framework**
University sponsorship as the dominant letterhead	Large University College London (UCL) logo placed at the top of the letter, with National Institute for Health Research (NIHR) logo placed at the bottom right.	Systematic review evidence [8, 9]
Salient and attractive letterhead to increase likelihood of attention and relevance	Coloured letterhead used (blue logo)	Salience (MINDSPACE) [22]
Authoritative messenger to convey importance and obligation	“I am the Co-Director of the Cancer Screening Group”	Messenger (MINDSPACE) [22]
Emphasising importance to elicit a sense of duty and personal value	“important research study “ “Your involvement is really valuable”	Ego (MINDSPACE) [22]
Referring to emotion to elicit personal connection	“how your test result has made you feel”	Affect (MINDSPACE) [22]
Conveying social norms by referencing the majority target group	“most women find…rewarding.” “result letters better for other women”	Norms (MINDSPACE) [22]
Language to convey personalisation	“I am interested in your particular test result” “I’d like to hear your views” “particularly interested in hearing from you”	Systematic review evidence [8, 9]
Perception of exclusivity and possible sanction (i.e., missing out)	“I am only inviting a select number...”	Ego and Incentive (MINDSPACE) [22]
Salience and visual breaking	Coloured subheadings (“Your role” and “Optional interview”) to break up paragraphs	Salience (MINDSPACE) [22]
Perceived sanction in bold to elicit loss-aversion	“You have three weeks...to take part”	Incentives (MINDSPACE) [22]
Minimise short-term costs (e.g., low effort) and emphasise gains	“easy and quick” “enjoyable and rewarding” “You just need to fill in… the short questionnaire”	Incentives (MINDSPACE) [22]
Assurance of confidentiality of survey answers	“your answers will be kept strictly confidential”	Systematic review evidence [8, 9]
Coloured written signature	A signature using bright blue ink at the end of the letter	Systematic review evidence [8]

Note: MINDSPACE refers to a behavioural science framework within the MINDSPACE Report [22]

**Fig. 2:**
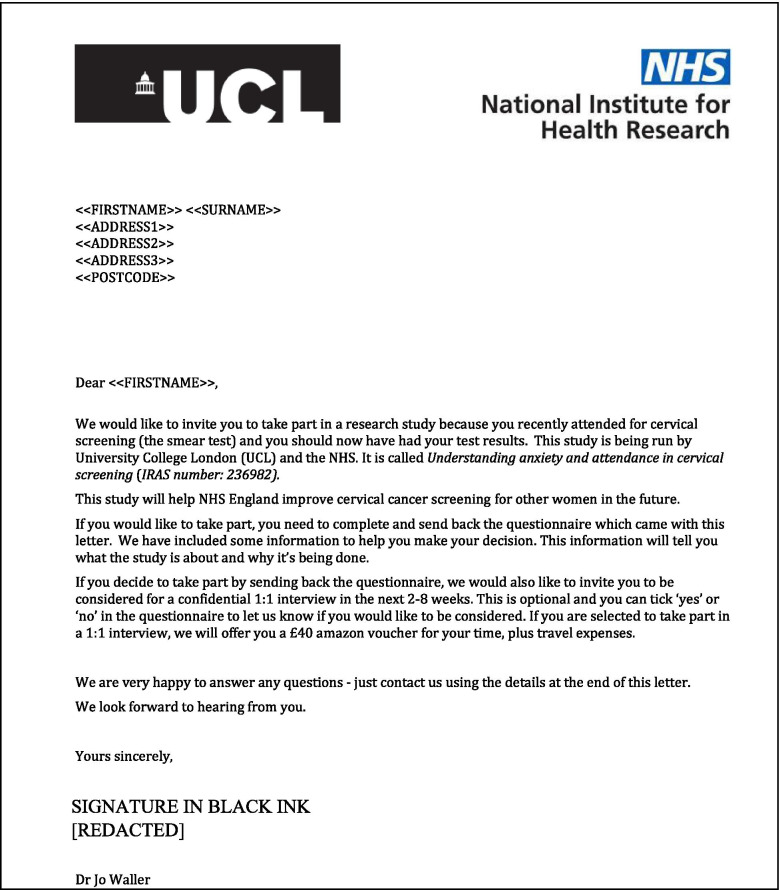
Cover letter used for the control group

In contrast, the control letter (see Fig. 2) was chosen to replicate similar standard wording suggested by the Health Research Authority (HRA), which is the regulatory body for research in NHS England [28].

The content used in both the intervention and control letters were drafted by a behavioural scientist (EM) and then discussed and iterated as part of a stakeholder engagement panel until there was consensus. The stakeholder panel consisted of eight individuals from a range of backgrounds including academia, policy, clinical practice, third sector, and patient and public representatives. The design of the other study materials (information sheet, survey) were pragmatically informed by standard practice recommended for NHS clinical studies, in line with our HRA ethical approvals and recommendations.

### Outcomes

The outcome was return of the survey (yes/no) within 3-months of the date of estimated screening test result delivery, which was the timeframe specified in the study protocol for the primary study.

### Demographic and clinical covariates

Covariates included demographic and clinical variables, which were prespecified due to their known or anticipated relationship with response rate [29–32]. Continuous covariate variables included age (years) and IMD Score (multidimensional marker of area-level deprivation based on residential postcode). Categorical covariates included NHS site, IMD Quintile, and screening test result (first HPV+/normal test result; or second or third consecutive HPV+/normal result at 12-month follow-up screen). The covariates were available for all participants (responders and non-responders) through access to clinical health records.

### Sample size

As this study was a nested trial, sample size estimates were based on the primary cross-sectional study [30], where the total sample size approached was 2702 women. Assuming participants were randomised equally (i.e., 1351 participants in each trial arm), and a baseline response rate of 21% (based on similar research [29]), the sample size for this study provided 80% power and a 5% margin for type II error to detect a between-group difference in response of at least 4.5% [33].

### Analysis

Statistical analyses were performed using Stata v15 and a *p*-value < 0.05 was considered statistically significant. Demographic characteristics were assessed descriptively and reported for the whole sample, and for responders and non-responders.

In the univariate analysis, logistic regression was used to ascertain whether survey response (yes/no) differed between the intervention letter vs. control letter. Logistic regression also tested whether survey response (yes/no) differed between clinical test result (1st vs. 2nd or 3rd consecutive HPV-positive with normal cytology result) and NHS site (North West London vs. Greater Manchester). Linear regression was used to assess the extent to which survey response (yes) was associated with age and IMD score.

Multivariate logistic regression was performed to assess whether survey response (yes/no) differed between the intervention vs. control letter, while adjusting for age, IMD score, NHS site, and test result.

Data completeness was > 95% for all variables except IMD score and IMD Quintile (94%). We used multiple imputation, using five iterations, to account for the missing IMD data and the model included the primary outcome and socio-demographic factors, which we assumed included all predictors of missingness. Data with > 95% completeness was treated as missing in the analysis. The final models were derived by fitting a regression model including all confounders, and estimates were combined using Rubin’s rules [34]. Sensitivity analysis was conducted comparing the complete dataset with the multiple imputed dataset, to check for differences in the results; there were no substantive differences. Results are presented using imputed data.

### Ethical approvals and trial registration

 HRA approval was granted on 09.01.2019 (Research Ethics Committee reference: 18/EM/0227 and Confidentiality Advisory Group reference: 18/CAG/0118). Cervical Screening Research Advisory Committee approval was granted on 15.03.2019 (ODR1819_005). Further details can be found on the ISRCTN clinical registration site (10.1186/ISRCTN15113095).

## Results

In total, 2702 individuals were invited to take part and mailed a survey; 1353 were randomised to the intervention and 1349 to the control arm. The mean age of the population was 37.5 years and the majority lived in the two most deprived IMD Quintiles in England (*n* = 1431, 56.2%; Quintiles 1 and 2). Around three quarters of participants were recruited through NHS Greater Manchester (*n* = 2090, 77.4%) and most had received their first HPV-positive with normal cytology screening result (*n* = 2202, 81.5%). Baseline characteristics were similar between the two randomised groups, with slight differences observed for some IMD quintiles (see Table 2).

**Table 2:** Demographic characteristics for the whole sample (overall and by intervention and control group) (N = 2702)

Variable	Control	Intervention	Total
**Total**	1349 (49.9%)	1353 (50.1%)	2702 (100%)
**Age (years)**			
Mean (SD)	37.4 (10.8)	37.6 (11.1)	37.5 (11.0)
Missing [n (%)]	1 (0.074%)	0 (0%)	1 (< 0.001%)
**IMD score**			
Mean (SD)	28.2 (18.2)	26.5 (16.5)	27.3 (17.4)
Missing [n (%)]	67 (5.0%)	85 (6.3%)	152 (5.6%)
**IMD quintile (*N*** **= 2550, 94.4%)**			
Quintile 1 (most deprived)	435 (33.9%)	374 (29.5%)	808 (31.7%)
Quintile 2	279 (21.8%)	344 (27.2%)	623 (24.5%)
Quintile 3	259 (20.2%)	236 (18.6%)	495 (19.4%)
Quintile 4	178 (13.9%)	173 (13.7%)	351 (13.8%)
Quintile 5 (least deprived)	131 (10.2%)	141 (11.1%)	271 (10.6%)
**NHS site (N = 2702, 100%)**			
Manchester	1043 (77.3%)	1047 (77.4%)	2090 (77.4%)
London	306 (22.7%)	306 (22.6%)	612 (22.6%)
**Cervical screening test result (N = 2702, 100%)**			
1st HPV+/normal cytology	1103 (50.1%)	1099 (49.9%)	2202 (81.5%)
2nd/3rd consecutive HPV+/normal cytology	246 (49.2%)	254 (50.8%)	500 (18.5%)

Note. *SD* standard deviation, *N *number of participants, %: percentage

Cervical screening test result was dichotomised to receiving a 1st HPV+/normal cytology test result vs. a 2nd or 3rd consecutive HPV+/normal cytology test result

**Fig. 3:**
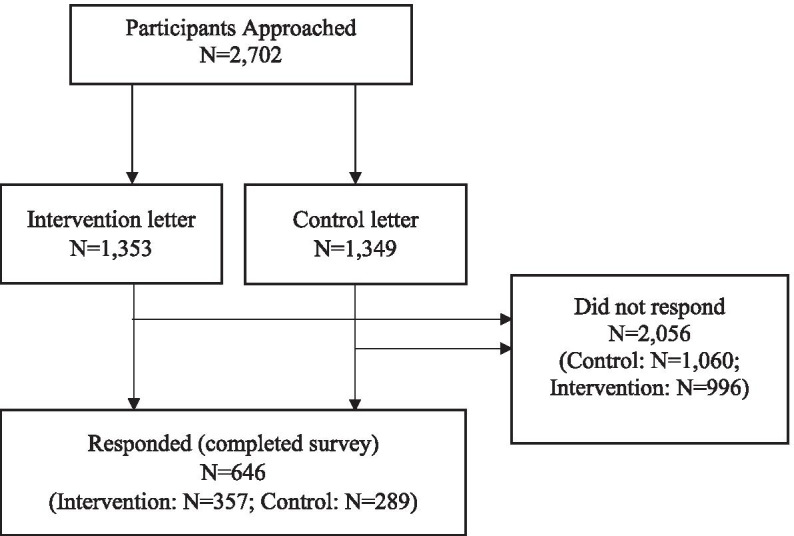
Flow diagram of participants approached and responders vs. non-responders

Overall, 646 participants returned the completed survey, generating a response rate of 23.9% (*n* = 357, 26.4%, intervention; *n* = 289, 21.4% control). Figure 3 displays a flow diagram of the recruitment process.

Table 3 presents demographic characteristics for responders (*n* = 646) and non-responders (*n* = 2056). Supplementary File 4 presents a table of demographic characteristics stratified by intervention vs. control group for responders.

**Table 3:** Demographic characteristics for responders and non-responders

	Responders (N = 646)	Non-Responders (N = 2056)
**Group allocation**		
Control	289 (44.7%)	1060 (51.6%)
Intervention	357 (55.3%)	996 (48.4%)
**Age (years)**		
Mean (SD)	38.3 (11.9)	37.3 (10.7)
Missing [n (%)]	0 (0%)	1 (0.0005%)
**IMD score**		
Mean (SD)	25.7 (16.0)	27.9 (17.7)
Missing [n (%)]	39 (6.0%)	113 (5.5%)
**IMD quintile**		
Quintile 1 (most deprived)	156 (25.7%)	653 (33.6%)
Quintile 2	176 (29.0%)	447 (23.0%)
Quintile 3	128 (21.1%)	367 (18.9%)
Quintile 4	88 (14.5%)	263 (13.5%)
Quintile 5 (least deprived)	59 (9.7%)	213 (10.9%)
**NHS site**		
Manchester	513 (79.4%)	1577 (76.7%)
London	133 (20.6%)	479 (23.3%)
**Test result**		
1st HPV+/normal cytology	505 (78.2)	1697 (82.5%)
2nd/3rd HPV+/normal cytology	141 (21.8%)	359 (17.5%)

Note: *SD *standard deviation, *N* number of participants, %: percentage

### Response in the intervention vs. control group (*n* = 2702)

Univariate analysis revealed higher odds of survey response in the intervention group (21.4 and 26.4% for the control and intervention group, respectively; OR 1.32, 95% CI: 1.10–1.57); those with lower IMD scores (less deprived; OR 0.99, CI: 0.99–1.00); and those with a 2nd or 3rd consecutive test result (22.9 and 28.2% for 1st and 2nd or 3rd result, respectively; OR 1.32, CI: 1.06–1.64). Participants who were older displayed higher odds of response (OR 1.01, CI: 1.00–1.02).

In the fully adjusted analyses, results were similar to the univariate analyses. We found significantly increased odds of returning a survey in the intervention group when compared with the control (aOR 1.30, CI: 1.09–1.55), in those with lower IMD scores (less deprived; aOR 0.99, CI: 0.99–1.00), and those who had received a 2nd or 3rd consecutive test result (aOR 1.29, CI: 1.04–1.61).

See Table 4 for an overview of the results.

**Table 4:** Univariate and multivariate regression results for survey response (yes) in the intervention vs. control and across demographics

Variable	Response (yes)	Unadjusted OR (95% CI)	*p*-value	Adjusted OR^a^ (95% CI)	*p*-value
**Group Allocation**					
Control	289 (21.42%)	–	0.003	–	0.004
Intervention	357 (26.39%)	1.32 (1.10–1.57)		1.30 (1.09–1.55)	
**Age (years)**					
Age	–	1.01 (1.00–1.02)	0.047	1.01 (1.00–1.02)	0.082
**Area-level deprivation**					
IMD score	–	0.99 (0.99–1.00)	0.011	0.99 (0.99–1.00)	0.017
**NHS Site**					
Manchester	513 (24.55%)	–	0.152	–	0.259
London	133 (21.73%)	0.85 (0.69–1.06)		0.88 (0.71–1.10)	
**Test result**					
Test result (1)	505 (22.93%)	–	0.013	–	0.024
Test result (2 + 3)	141 (28.2%)	1.32 (1.06–1.64)		1.29 (1.04–1.61)	

Note: Test result (1): 1st HPV+/normal cytology, Test result (2 + 3): 2nd or 3rd HPV+/normal cytology, OR: odds ratio, CI: confidence interval, %: percentage

^a^Adjusted for IMD score, age, NHS site, and test result

## Discussion

Almost all postal questionnaire studies incorporate an invitation or cover letter. We found that applying behavioural science and evidence-based methods to routine invitation letters improved postal response to a health-related survey, after adjusting for demographic and clinical characteristics. As survey participation rates continue to decline worldwide [4–6], our findings provide support for the pragmatic and cost-effective adoption of combined techniques in routine research to increase postal response rates.

Consistent with previous systematic reviews evaluating the application of individual techniques, we found that combining several techniques positively influenced postal response rate [1, 7–10]. The magnitude of effect observed in our study (adjusted odds ratio of 1.30 in favour of the intervention) is higher than found in some isolated techniques which similarly carry low or minimal financial costs, such as adopting personalisation or use of non-monetary incentives (odds ratios of 1.16 and 1.13, respectively [2, 9]). However, this is not the case when compared to all cost-effective isolated techniques, such as mentioning an obligation to respond or the use of university sponsorship, which demonstrate similar or slightly larger effects (odds ratios of 1.61 and 1.32, respectively [8, 9]). Furthermore, our approach appeared to yield a lower effect size than certain more financially expensive or resource-intensive strategies, such as providing monetary incentives, use of recorded mailed delivery, and pre-notifying participants (odds ratios of 1.87–1.99, 1.76–2.04, and 1.45–1.50, respectively [8, 9]).

Ultimately, however, findings which are based on isolated techniques cannot act as a direct comparator to our study. This is partly due to differences in the content used in the control arms of studies and variations in adjustments for confounders and contexts. For example, in our study, the control and intervention letters both utilised some techniques which have been shown to increase participant response, such as providing assurance of confidentiality and a conditional incentive of financial payment for participation in an interview [8, 9]. Similarly, we provided a second copy of our questionnaire at follow-up which has been shown to improve response [9]. Utilising these evidence-based techniques in our control letter mirrors standard research practice; however, this differs to several previous studies which avoid using techniques in control conditions or do not report control conditions. It is therefore possible that the effect sizes observed in our study could be subject to ceiling effects or reflective of additive effects. Conversely, our study questionnaire (sent to all participants) asked about a sensitive health topic and our study information sheet explicitly stated that participants could opt out, which have both been found to reduce the odds of response [8, 9]. Hence, overall, these heterogeneities in methodology and study contexts prohibit comparative conclusions relative to the previous literature.

Using a combination of techniques in our study also introduces the possibility of interaction and/or moderation effects between individual techniques, which we could not measure or test. Two or more techniques implemented in tandem may have led to differential impacts on response rate, when compared with the same techniques used in isolation. Hence, a core limitation of our pragmatic approach is that we are unable to determine optimal combinations of techniques and, similarly, whether certain combinations may have reduced response or counteracted positive effects. Further investigation is needed to test the magnitude of effects using different combinations of techniques (e.g., through adopting a factorial RCT design) and to assess the impact of potential interactions.

Response rate is known to be influenced by sociodemographic factors such as age, sex, educational attainment, ethnicity, marital status, and deprivation [5, 31, 32, 35, 36]. Living in a less deprived area (lower index of multiple deprivation score) yielded a small statistically significant effect size in favour of returning a survey in our study (adjusted odds ratio of 0.99), but we observed no effect for age in our adjusted analyses. We did not test for interaction effects between area-level deprivation and response to the survey, as this was not part of our planned analysis and due to the likelihood of issues with statistical power. Also, we did not have data on other important sociodemographic variables like ethnicity and education. Hence, even though our intervention increased survey response overall whilst adjusting for some demographic factors, we cannot rule out that bias remains for particular sociodemographic groups.

Improving response rates in survey-based studies remains a priority for health and epidemiological research. It is hoped that the gains yielded from better sample representativeness and lower non-response bias should ultimately translate into improved public and patient outcomes [37]. Implementation of behavioural science techniques in routine research practice may offer a low-cost solution for generating higher response rates and thus enhancing quality of care.

### Limitations

Our study carries several limitations. Although our intervention was found to increase response to a health survey, the overall response rate remained low (23.9%). This may reflect selection bias in our sample, which could lead to an over- or- underestimation of the intervention effect when compared with the general population. Furthermore, our target population only included women attending cervical screening, limiting the applicability of our findings to more general health contexts and to men. Some research has indicated that women are more likely to respond to research studies than men [31, 36], therefore, it is possible that there may also be moderation effects for gender in interventions targeting response rates. We also only recruited through two clinical sites in England which, although covering large geographical regions, may affect the generalisability of our findings, especially when compared with other cultural or sociodemographic contexts. Lastly, as this was a nested trial within a cross-sectional survey study, our target sample size was based on the primary cross-sectional study; the sample size calculation reported in this RCT was post-hoc. Although we were appropriately powered for the main analysis, we were unable to test for potentially relevant interaction or moderation effects due to the likelihood of being underpowered.

## Conclusion

Using a combination of easy-to-implement behavioural science and evidence-based techniques in a study invitation letter increased participant response to a health survey. The major benefit of this pragmatic approach was the absence of substantive additional research costs, like providing financial incentives or additional follow-up mailing strategies. Further research is needed to investigate the optimal combinations of techniques for increasing postal response.

## Supplementary Information

**Additional file 1. **CONSORT 2010 checklist of information to include when reporting a randomised trial*.

**Additional file 2.** The TIDieR (Template for Intervention Description and Replication) Checklist*.

**Additional file 3. **Survey used in the study. 

**Additional file 4: Supplementary File 4.** Demographic characteristics for responders by intervention vs. control group (*N* = 646).

## Data Availability

The datasets used and/or analysed during the current study are available from the corresponding author on reasonable request.

## Abbreviations

CI: Confidence Interval
CONSORT: Consolidated Standards of Reporting Trials
HPV: Human papillomavirus
IMD: Index of multiple deprivation
ISRCTN: International Standard Randomised Controlled Trial Number
MINDSPACE: Messenger; Incentive; Norms; Default; Salience; Priming; Affect; Commitment; Ego
NHS: National Health Service
OR / aOR: Odds Ratio/ Adjusted Odds Ratio
RR: Response Rate
TIDieR: Template for Intervention Description and Replication
UCL: University College London
