# Supplementary material for: Improving postal survey response using behavioural science: a nested randomised control trial
Source: BMC Med Res Methodol. 2021 Dec 18;21:280. doi: 10.1186/s12874-021-01476-7 (PMC8684081; doi:10.1186/s12874-021-01476-7)
Supplement: Supplementary file 3 — Additional file 3. Survey used in the study. [file 12874_2021_1476_MOESM3_ESM.pdf]

UCL

TODAY'S DATE: ___________________________

Can you remember what your most recent cervical screening result was?
You may have had an HPV test result or a cytology (smear) test result or both

HPV test			
☐ HPV was found	☐ HPV was not found	☐ No HPV test	☐ Not sure
**Cytology (smear) test**			
☐ Normal cytology (no cell changes)	☐ Abnormal cytology (cell changes found)	☐ No cytology test	☐ Not sure

First, we'd like to know how you're feeling at the moment

Please read each statement below and tick the best box that indicates the way you feel right now

	Not at all	Somewhat	Moderately	Very much so
I feel calm	☐	☐	☐	☐
I feel tense	☐	☐	☐	☐
I feel upset	☐	☐	☐	☐
I feel relaxed	☐	☐	☐	☐
I feel content	☐	☐	☐	☐
I feel worried	☐	☐	☐	☐

Since my cervical screening (smear) result I have been feeling…

	Strongly agree	Agree	Disagree	Strongly disagree
In good general health	☐	☐	☐	☐
Happy about the way my body feels	☐	☐	☐	☐
In control of my body	☐	☐	☐	☐
Worried that I may have something seriously wrong	☐	☐	☐	☐
Concerned about my fertility	☐	☐	☐	☐
In good gynaecological health	☐	☐	☐	☐
Fearful about cervical cancer	☐	☐	☐	☐
Interested in sex	☐	☐	☐	☐
Optimistic about my future health	☐	☐	☐	☐

How concerned do you feel about your screening result?

Not at all concerned	Slightly Concerned	Somewhat concerned	Moderately concerned	Very concerned
☐	☐	☐	☐	☐

How much do you agree with the following statements?

	Strongly disagree	Disagree	Agree	Strongly agree
I am concerned about cervical cancer	☐	☐	☐	☐
I am concerned about my sex life	☐	☐	☐	☐
I am concerned about the impact of my screening result on my partner	☐	☐	☐	☐
I am concerned about something else	☐	☐	☐	☐

If you are concerned about something else, please indicate what:

____

____

____

If you are concerned, please indicate what you are <u>most</u> concerned about? Please ONLY tick one.

- ☐ Cervical cancer
- ☐ My sex life
- ☐ The impact of my screening result on my partner
- ☐ Something else
- ☐ Not applicable - I am not concerned

Have you told anyone about your screening result?

☐ Yes ☐ No ☐ I'd rather not say

If yes, who did you tell?

Partner	Friend	Family	Health professional	Someone else (please indicate who)
☐	☐	☐	☐	- -

**Thinking about what HPV means to you (please circle on a scale 1-10)…**											
How much does having HPV affect your life?	0	1	2	3	4	5	6	7	8	9	10
	No affect at all										*Severely affects my life*
How much longer do you think you will have HPV?	0	1	2	3	4	5	6	7	8	9	10
	A very short time										*Forever*
How much control do you feel you have over your HPV?	0	1	2	3	4	5	6	7	8	9	10
	Absolutely no control										*Extreme control*
How much do you think cervical screening can help?	0	1	2	3	4	5	6	7	8	9	10
	Not at all helpful										*Extremely helpful*
How much do you experience symptoms from your HPV?	0	1	2	3	4	5	6	7	8	9	10
	No symptoms at all										*Extreme symptoms*
How concerned are you about your HPV?	0	1	2	3	4	5	6	7	8	9	10
	Not at all concerned										*Extremely concerned*
How well do you feel you understand HPV?	0	1	2	3	4	5	6	7	8	9	10
	Don't understand at all										*Understand very clearly*
How much does having HPV affect you emotionally?	0	1	2	3	4	5	6	7	8	9	10
	Not at all affected										*Extremely affected*

Please list in order the three most important things that you believe caused your HPV
The most important causes for me are…

1. ____
2. ____
3. ____

You will be invited back to cervical screening in around 12 months. Will you attend?

Yes, definitely	Yes, probably	Probably not	Definitely not
☐	☐	☐	☐

Is there anything that might stop you from attending your next cervical screen (smear test)?

____

____

____

Do you think any of the things below might stop you from attending your next screen (smear test)?
Please tick all that apply.

- ☐ No, nothing will stop me
- ☐ I might forget
- ☐ I don't like being screened
- ☐ I find screening painful
- ☐ I find screening embarrassing
- ☐ It's difficult to make an appointment at a time that suits me
- ☐ I don't want to find out my test result
- ☐ I am at low risk of cervical cancer
- ☐ I might be too busy
- ☐ It's not my main priority

Have you had any symptoms recently that you think might be related to your HPV? Please list any symptoms.

____

____

____

We've listed a number of general symptoms that you may or may not have experienced in the last 4 weeks…

	I have experienced this symptom within the last 4 weeks		If yes, I think this symptom is related to my HPV	
	Yes	No	Yes	No
Pain	☐	☐	☐	☐
Sore throat	☐	☐	☐	☐
Nausea	☐	☐	☐	☐
Unusual bleeding	☐	☐	☐	☐
Breathlessness	☐	☐	☐	☐
Weight loss	☐	☐	☐	☐
Vaginal discharge	☐	☐	☐	☐
Fatigue	☐	☐	☐	☐
Stiff joints	☐	☐	☐	☐
Sore eyes	☐	☐	☐	☐
Pain during sex	☐	☐	☐	☐
Wheeziness	☐	☐	☐	☐
Headaches	☐	☐	☐	☐
Upset stomach	☐	☐	☐	☐
Weight gain	☐	☐	☐	☐
Sleeping difficulties	☐	☐	☐	☐
Dizziness	☐	☐	☐	☐
Loss of strength	☐	☐	☐	☐

Now please think about when you feel particularly stressed. Do you tend to…

	Not at all	A little bit	A fair amount	A lot
Turn to work or other activities to take your mind off things	☐	☐	☐	☐
Get emotional support from others	☐	☐	☐	☐
Give up trying to deal with the situation	☐	☐	☐	☐
Do something to try to make the situation better	☐	☐	☐	☐
Visit your GP	☐	☐	☐	☐
Refuse to believe what is happening	☐	☐	☐	☐
Try to see the situation in a different light, to make it seem more positive	☐	☐	☐	☐
Look for extra information	☐	☐	☐	☐
Try to come up with a strategy about what to do	☐	☐	☐	☐
Learn to live with the situation	☐	☐	☐	☐
Criticise yourself	☐	☐	☐	☐
Speak to your partner, friends or family about it	☐	☐	☐	☐
Use alcohol or other drugs to help you get through it	☐	☐	☐	☐
Pray or meditate	☐	☐	☐	☐
Make fun of the situation	☐	☐	☐	☐

Do you have any comments about your NHS cervical screening test result letter or information leaflet that you received?

____

____

____

How could the NHS improve their screening result letters or information leaflets?

____

____

____

The NHS are thinking about ways they could inform women about their cervical screening test results in the future. Please tick <u>all</u> the ways you would be happy to receive your test results.

Letter	Text message	Email	Mobile app
☐	☐	☐	☐

Finally, we'd like to ask you a few general background questions

How old are you? ____________________

Please tick the box that best describes your ethnic group

- ☐ White (British or Other)
- ☐ Black/African/Caribbean/Black British
- ☐ Mixed/multiple ethnic groups
- ☐ Asian/Asian British
- ☐ Other ethnic group
- ☐ Prefer not to say

Are you currently diagnosed with an anxiety disorder or depression (or have you been previously)?

	Currently diagnosed			Previously diagnosed		
	Yes	No	Prefer not to say	Yes	No	Prefer not to say
Anxiety disorder	☐	☐	☐	☐	☐	☐
Depression	☐	☐	☐	☐	☐	☐
Other mental health diagnosis: ________________________________	☐	☐	☐	☐	☐	☐

Do you know anyone who has ever been diagnosed with cervical cancer?

- ☐ No
- ☐ Yes, somebody close to me
- ☐ Yes, somebody I know but we're not very close

What is the highest level of education you have completed? (Please tick one only)

- ☐ Degree or higher degree
- ☐ Higher education qualification
- ☐ Below degree level
- ☐ A-levels
- ☐ ONC/BTEC
- ☐ O-Levels
- ☐ No formal qualifications

How would you describe your current relationship status? (Please tick one only)

- ☐ Single
- ☐ In a relationship
- ☐ Living with partner
- ☐ Married/in a civil partnership
- ☐ Widowed
- ☐ Divorced
- ☐ Other (please specify): ___________________________________

Thank you for taking the time to complete this questionnaire.

PLEASE RETURN THIS QUESTIONNAIRE TO UCL USING THE PRE-PAID ENVELOPE PROVIDED.
