# Supplementary material for: Improving postal survey response using behavioural science: a nested randomised control trial
Source: BMC Med Res Methodol. 2021 Dec 18;21:280. doi: 10.1186/s12874-021-01476-7 (PMC8684081; doi:10.1186/s12874-021-01476-7)
Supplement: Supplementary file 4 — Additional file 4: Supplementary File 4. Demographic characteristics for responders by intervention vs. control group (N = 646). [file 12874_2021_1476_MOESM4_ESM.docx]

**Supplementary File 4.** Demographic characteristics for responders by intervention vs. control group (N=646)

| Variable | Control | Intervention | Test* |
| --- | --- | --- | --- |
| **Total (N)** | 289 | 357 |  |
| **Age (years)** |  |  |  |
| Mean (SD) | 38.0 (11.6) | 38.4 (12.1) | t=-0.44; p=0.660 |
|  |  |  |  |
| **IMD scores** |  |  |  |
| Mean (SD) | 26.7 (17.0) | 24.8 (15.1) | t=1.42; p=0.157 |
| Missing | 10 (3.5%) | 29 (8.1%) |  |
| **IMD quintile** |  |  |  |
| Quintile 1 (most deprived) | 81 (29.0%) | 75 (22.9%) | X^2^=9.53; p=0.049 |
| Quintile 2 | 67 (24.0%) | 109 (33.2%) |  |
| Quintile 3 | 62 (22.2%) | 66 (20.1%) |  |
| Quintile 4 | 46 (16.5%) | 42 (12.8%) |  |
| Quintile 5 (least deprived) | 23 (8.2%) | 36 (11.0%) |  |
| **NHS site** |  |  |  |
| Manchester | 229 (79.2%) | 284 (79.6%) | X^2^=0.01; p=0.922 |
| London | 60 (20.8%) | 73 (20.4%) |  |
| **Test result** |  |  |  |
| 1st HPV+/normal cytology | 218 (75.4%) | 287 (80.4%) | X^2^=2.30; p=0.129 |
| 2nd/3rd HPV+/normal cytology | 71 (24.6%) | 70 (19.6%) |  |

Note. SD: standard deviation, N: number of participants, %: percentage.

*This study was not powered to test for differences in demographic characteristics between the intervention and control groups; therefore, caution is warranted in the interpretation of the significance testing presented in this table.
